# Sigmoid Colon Intussusception Secondary to Giant Colonic Submucosal Lipoma: A Case Report

**DOI:** 10.1155/carm/9948237

**Published:** 2025-05-21

**Authors:** Mohammed Alessa

**Affiliations:** Department of Surgery, College of Medicine, King Faisal University, Al Hofuf, Saudi Arabia

## Abstract

**Background:** Lipoma is one of the benign tumors that originate from adipose tissue, most likely in the neck, chest, back, shoulders, arms, and thighs. It is rare to find lipoma originating from submucosal adipose tissue. Colonic submucosal lipomas develop at frequency of 0.035%–4.4%. The incidence of submucosal colonic lipoma is 0.15% at colonoscopy. Intussusception is a common cause of bowel obstruction in children; however, it is rare in adults. Usually, it has a malignant background in adults.

**Case Presentation:** A 43-year-old male presented to the hospital with a history of intermittent abdominal pain for 6 months. Pain is associated with alternating diarrhea and constipation. Physical examination showed left lower abdominal tenderness. CT scan of the abdomen showed sigmoid colo-colonic intussusception.

**Discussion:** Colo-colonic intussusceptions account for 17% of all intestinal intussusceptions in adults, and it is most likely caused by malignant lesions rather than a submucosal lipoma.

**Conclusion:** Submucosal lipoma is a rare cause of colo-colonic intussusceptions. It should be considered in differential diagnosis.

## 1. Introduction

Lipoma is defined as a nonepithelial benign adipose tumor that appears most commonly in the neck, chest, back, shoulders, arms, and thighs. It is very rare to find lipoma originating from submucosal adipose tissue. Although it is rare, the most common gastrointestinal tract submucosal lipoma is located in the colon [1]. Colonic submucosal lipomas develop at frequency of 0.035%–4.4% [2, 3]. They are most commonly present as sessile polyps rather than pedunculated with a wide range of dimensions from 2 mm to 30 cm. Majority of lipomas are asymptomatic and discovered incidentally during colonoscopy, surgery, or autopsy but some of them may present with abdominal pain, diarrhea, constipation, or picture of intestinal obstruction mimicking colon cancer and rarely intussusception [4, 5].

Intestinal intussusception is defined as the invagination of an intestinal loop with a mesenteric fold (intussusceptum) in the lumen of a continuous portion of the intestine (intussuscipiens) following peristalsis [1]. Intestinal intussusception is a common cause of bowel obstruction in children, and it accounts for less than 1% of adults. Most cases occur in the ileocecal area or small bowel rather than the colon. Colo-colonic intussusception accounts for 17% of all gastrointestinal intussusception. Malignant lesions most commonly cause adult Colo-colonic intussusception and are less likely due to inflammatory diseases, adhesions, and other mechanical diseases affecting peristalsis and leading to motility disorders [6]. The most common malignant causes leading to colo-colonic intussusception are adenocarcinomas and lymphoma [7]. In this article, I present a colo-colonic intussusception case caused by submucosal pedunculated lipoma.

## 2. Case Presentation

**Figure 1:**
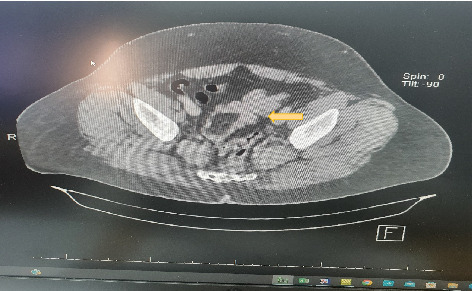
CT scan abdomen showed proximal colon dilatation with distal collapse.

**Figure 2:**
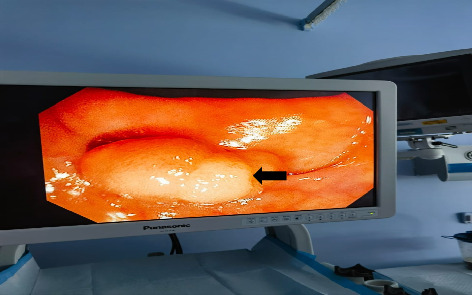
Colonoscopy showed intraluminal appearance of submucosal lipoma.

**Figure 3:**
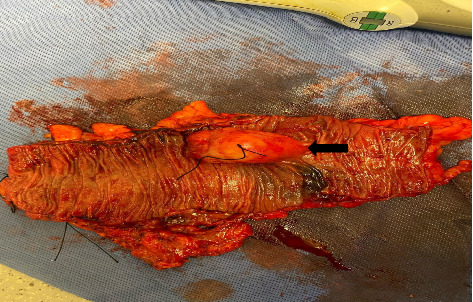
Resected specimen showed submucosal swelling around 3∗5 cm.

A 43-year-old male not known to have any chronic medical illness presented to emergency department with recurrent attacks of left lower quadrant abdominal pain for 6 months with several visit to emergency department. Pain is localized in the left iliac fossa and associated with alternating diarrhea and constipation. In the last 10 days prior to the presentation, he developed bleeding per rectum. It was fresh blood of a little amount mixed with stool. There is no history suggestive of bowel obstruction. Examination showed a 43-year-old male with a good general appearance, hemodynamically stable, with mild tenderness on the left iliac fossa and no piles, fissures, or anal masses. Laboratory tests are within normal range. Abdominal ultrasonography was not done. CT scan of the abdomen with oral and intravenous contrast showed sigmoid colo-colonic intussusception with a leading point. The swelling measures 5∗3.6∗2.50 cm with the possibility of lipoma (Figure 1). A colonoscopy was done that showed a sigmoid pedunculated whitish lesion that measures about 3∗5 cm (Figure 2). The patient was prepared for surgery during the last hospitalization after confirmation of diagnosis and optimization of his condition without any trial of reduction. He underwent an uneventful laparoscopic sigmoidectomy with primary anastomosis (Figure 3). The postoperative course was smooth without any complications. He was discharged home after 4 days. The final histopathology showed a morphologic picture suggestive of lipoma with overlying mucosal ulceration, inflammation, and granulation tissue formation with eight reactive lymph nodes and no dysplasia or malignancy in the examined specimen.

## 3. Discussion

Colonic lipoma is the third most common benign tumor of the large bowel after hyperplastic and adenomatous polyps, with incidence ranging between 0.035% and 4.4% [8]. Most colonic lipomas are small, asymptomatic, and discovered incidentally. However, colonic lipomas can present with clinical manifestations, including colono-colonic intussusception [9]. Colonic lipomas are common in females between the ages of 50–60 years, most commonly located in the ascending colon followed by the descending, transverse colon and rectum [10]. Majority of lipomas are asymptomatic and discovered incidentally during colonoscopy, surgery, or autopsy. However, lipomas larger than 5 cm are considered giant and are symptomatic. The most common symptoms are abdominal pain, bleeding, colonic obstruction, and intussusceptions.

Intestinal intussusception is a common cause of bowel obstruction in children, and it accounts for less than 1% in adults [11]. Based on site, intussusception is divided into four main types: entero-enteric, colo-colonic, ileocolic, and ileocecal [11, 12]. Initial diagnosis is difficult, especially when it comes to distinguishing colonic lipoma from malignancy. Abdominal ultrasonography can be used initially for diagnosis, although it is an operator dependent and needs a skilled radiologist, especially if the lipoma is less than 2 cm [13]. CT scan is helpful in the diagnosis of colonic lipoma. MRI can visualize lipoma better than other image modalities with the use of different tissue intensities. Colonoscopy can provide direct visualization of lipoma with the possibility of obtaining tissue biopsy [11, 13].

Adult intussusception requires resection, especially if it is greater than 2 cm, preoperative diagnosis is uncertain, or there is a suspension of malignancy [11]. Resection can be done by endoscopy or segmental resection through open or laparoscopic techniques. If the endoscopic approach is not feasible, segmental resection can be done. The laparoscopic approach is preferred due to cosmetic and early postoperative recovery [14].

## 4. Conclusion

Intestinal intussusception is a common cause of bowel obstruction in children, but it is rare in adults. Preoperative diagnosis can be difficult although there are different investigation tools. Almost consider surgical resection of adult intussusception because malignancy is the main causative factor, while submucosal lipoma accounts for small numbers. Further research is needed for a better understanding of disease pathology and to enrich the literature for better management of such conditions [15, 16].

## Data Availability

The data that support the findings of this study are available on request from the corresponding author. The data are not publicly available due to privacy or ethical restrictions.
